# Nicotine’s impact on platelet function: insights into hemostasis mechanisms

**DOI:** 10.3389/fphar.2024.1512142

**Published:** 2025-01-20

**Authors:** Xiayu Wu, Yongjun Liu, Changhao Zou, Fuqin He, Fang Guo, Sijia Liu, Yi Fan, Xuedong Zhu, Qianyi Zhou, Dan Shu

**Affiliations:** ^1^ Institute of Pharmaceutical Innovation, Hubei Province Key Laboratory of Occupational Hazard Identification and Control, School of Medicine, Wuhan University of Science and Technology, Wuhan, China; ^2^ Institute of Cardiovascular Diseases, Hubei Province Key Laboratory of Occupational Hazard Identification and Control, School of Medicine, Wuhan University of Science and Technology, Wuhan, China; ^3^ Hunan Tobacco Science Research Institute, Changsha, China

**Keywords:** nicotine, platelets, thrombin, [Ca^2+^]i, PAR4 receptor pathway, αIIbβ3

## Abstract

**Introduction:**

Traditional Miao and Dai Chinese medicines have used nicotine-rich leaf tobacco to treat traumatic injuries by promoting hemostasis. While nicotine is known to enhance platelet aggregation, its effects on other platelet functions and underlying mechanisms remain unclear.

**Methods and Results:**

This study aimed to thoroughly investigate nicotine’s effects on human platelets and its pharmacological mechanisms, using thromboelastography to assess nicotine’s impact on platelet function during coagulation. This study aimed to investigate the functional effects of nicotine on human platelets and elucidate its pharmacological mechanisms. The impact of nicotine on platelet function during the coagulation process was assessed using thromboelastography. Further studies showed that nicotine fully activates washed platelets, promoting aggregation, granule release, adhesion, spreading, and plaque retraction. Concurrently, nicotine was found to enhance the intracellular concentration of calcium ions in platelets ([Ca^2+^]_i_). To explore the underlying mechanisms, molecular docking software was employed to identify the platelet membrane receptors PAR1 and PAR4, which exhibited the highest docking scores with nicotine. Intervention with two receptor inhibitors demonstrated that only the PAR4 inhibitor could reverse the stimulatory effects of nicotine on platelet granule release. Through the examination of alterations in the downstream signaling pathways of PAR4 receptors, it was determined that nicotine promo-facilitates the phosphorylation of PI3K, AKT, and ERK1/2 proteins, subsequently contributing to the activation of αIIbβ3 receptors in platelets. Conversely, the application of PAR4 inhibitors was found to reverse these effects.

**Discussion:**

In conclusion, nicotine activates αIIbβ3 receptors and significantly enhances platelet function by promoting the phosphorylation of the platelet PAR4 receptor signaling pathway. These findings suggest the potential utility of nicotine as a hemostatic agent.

## 1 Introduction

Hemostasis is a complex physiological process involving vasoconstriction, platelet thrombosis, and blood coagulation (Zuhayri et al., 2023). Nicotine, the primary alkaloid in tobacco leaves, has demonstrated potential applications in the medical field (Okita et al., 2016). Nicotine can trigger the release of dopamine, producing pleasurable and addictive effects. Additionally, it can modulate the cardiovascular system, leading to elevated blood pressure and increased heart rate (Benowitz and Burbank, 2016). Studies have also shown that nicotine has neuroprotective, cognitive-enhancing, and anti-inflammatory effects (Levin et al., 2006; Beer, 2016). In recent years, there has been growing research interest in the non-traditional pharmacological roles of nicotine, particularly its potential therapeutic effects in neurodegenerative diseases like Alzheimer’s and Parkinson’s (Liu et al., 2007; Yang et al., 2023; Cao et al., 2024). It can serve as a drug intermediate for cardiovascular, neurological, and metabolic medications (Kim et al., 2000; Sood et al., 2011). Additionally, various traditional medicines, such as Miao, Dai, and Hani medicine, utilize tobacco leaves for treating cuts and burns, indicating the hemostatic properties of tobacco leaves when used externally. Platelets, small anucleate cell fragments with a disc-like shape, are essential in physiological and pathological processes such as hemostasis, wound healing, and thrombosis (Shakeri Shamsi and Taheri Soodejani, 2024). When blood vessels are damaged, platelets adhere to the vessel wall (Montenont et al., 2016), promoting thrombin production and forming platelet plugs, thereby aiding in hemostasis. The impact of nicotine on hemostasis is multifaceted, as it can modulate the coagulation process through various mechanisms; it can influence the coagulation process by affecting vascular contraction (Kuhlmann et al., 2005), altering endothelial cell function (Egleton et al., 2009), regulating the secretion of growth factors (Cirillo et al., 2006) and modulating platelet activity (Anindita et al., 2024). Additionally, nicotine may indirectly affect hemostasis by altering inflammatory responses. Recent studies have shown that nicotine can promote platelet aggregation and affect the activity (Yin and Rubenstein, 2013) and expression of angiotensin-converting enzyme (Ljungberg and Persson, 2008), impacting the thrombosis process. Nonetheless, the precise mechanism by which nicotine influences platelet function remains unclear. This study aims to comprehensively explore the effect of nicotine on platelet function (including aggregation, granule release, αIIbβ3 activation, adhesion expansion, and plaque retraction). This study aims to elucidate the underlying mechanisms by which nicotine influences platelet behavior through a detailed examination of these processes.

## 2 Materials and methods

### 2.1 Reagents and chemicals

**FIGURE 1:**
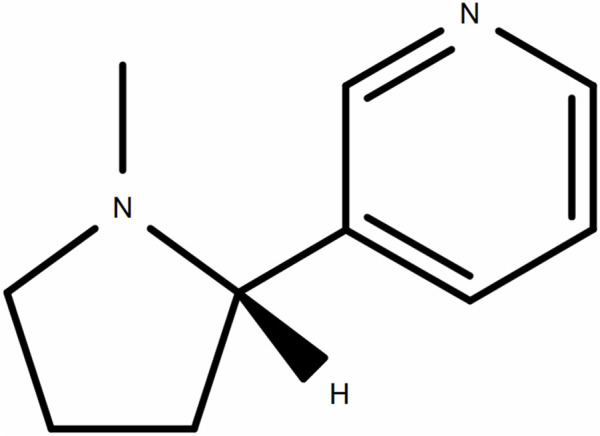
Chemical structure of nicotine.

Nicotine (high-performance liquid chromatography grade with ≥98%; Figure 1) was purchased from Aladdin (Shanghai, China). It was dissolved in normal saline (NS) to prepare a working solution with a final concentration of 5 mM throughout the experiments. Thrombin, prostaglandin E1 (PGE1), bovine serum albumin (BSA), Fluorescein isothiocyanate (FITC)-labeled phalloidin, EDTA and tetramethylrhodamine isothiocyanate (TRITC)-labeled phalloidin were purchased from Sigma Chemicals (St. Louis, MO). The antibodies against phospho-AKT (Ser473), phospho-PI3K (Tyr458), phospho-ERK1/2 (Tyr202/204), total-AKT, total-PI3K, and total- ERK1/2 were purchased from Cell Signaling (Beverly, MA). Biolegend (San Diego, CA, United States) supplied the FITC-conjugated anti-P-selectin (CD62P) and FITC-conjugated anti-PAC-1 antibodies. Fluo-3 AM was obtained from MCE (Shanghai, China). For thromboelastometry assays, recombinant human thromboplastin (Dade-Innovin) was purchased from DRNX-III, Dingrun Medical Equipment Corp (Chongqing, China). All other chemicals used in the current study were of analytical reagent grade.

### 2.2 Study subjects

Obtaining washed human platelets for our research was done by collecting 20 mL of whole blood from volunteer college students between the ages of 18 and 25, both genders. Participants were required to be free of drugs and abstain from any substances that could influence their platelet function (e.g., alcohol, caffeine, aspirin and so on) for a minimum of 2 weeks prior to the collection, and to have no habits of excessive alcohol consumption, smoking, or other addictive behaviors. Prior to the collection process, we ensured that informed consent was secured from each participant, and all procedures involving human subjects and blood samples were conducted in accordance with ethical standards. The research adhered to the Helsinki Declaration’s guidelines and garnered the green light from the Wuhan University of Science and Technology’s Ethics Board (No. 2022158, September 2022).

### 2.3 Thromboelastometry assay

Utilizing a minuscule amount of tissue factor, whole blood thromboelastometry (minTF TEM) was initiated with a diluted form of human recombinant thromboplastin, reaching a final dilution ratio of 1–7,200. During each trial, a blend of 330 μL of whole blood was combined with 20 μL of 0.2 mol/L calcium chloride (CaCl_2_) and 10 μL of the aforementioned diluted thromboplastin, along with saline as vehicle control and nicotine (10, 20, 50 μM) as the drug intervention group, which were all added at the beginning of the experiment. After a 5-min incubation, detection was initiated within the ROTEM analysis cups using a ROTEM^®^ delta thromboelastograph system. The assessment process commenced 2 s post-mixing, and a suite of thromboelastometric metrics was ascertained, including Clotting Time (CT), Clot Formation Time (CFT), α-angle, and Maximum Clot Firmness (MCF). CT refers to the interval from the launch of the sample analysis to the emergence of the first discernible clot, marked by an amplitude of 2 mm. CFT spans from the CT to the moment when the clot’s firmness hits a benchmark of 20 mm. The α-angle quantifies the dynamics of clot formation, while MCF denotes the peak vertical deflection observed in the thromboelastometry graph, indicative of the clot’s ultimate strength and stability. The data collection process was permitted to extend up to a maximum of 2 hours.

### 2.4 Preparation of washed human platelets

Blood samples were drawn from a pool of healthy participants. These samples were treated with a 3.8% sodium citrate mixture at a 9:1 volume-to-volume ratio to prevent clotting and then spun at 950 revolutions per minute for 14 min to isolate the platelets. Post-separation, the platelets were rinsed twice and reconstituted in Tyrode’s buffer—a solution containing 13.8 mM NaHCO_3_, 13.7 mM NaCl, 0.36 mM NaH_2_PO_4_, 2.5 mM KCl, 5.5 mM glucose, and 20 mM HEPES, all balanced to a pH of 7.4. The resulting platelet concentration was standardized to 3 × 10^8^ platelets/mL (Zhu et al., 2022). The entire preparation procedure was carried out at ambient temperature. Prior to application, the cleaned platelets were left to sit at room temperature for 30 min.

### 2.5 Cytotoxicity assay

Wash platelets and adjust to a concentration of 3 × 10^8^ platelets/mL. The washed platelet suspension was dispensed into EP tubes and CaCl_2_ solution was added to give a final concentration of 1 mM. Different groups were set up and labelled, including background blank wells (Tyrode’s buffer, no cells), sample control wells (untreated control cells without drug treatment), toxicity maximal wells (untreated control wells used for detection of maximal toxicity), and drug-treated wells (including a drug concentration gradient). Two parallel duplicate wells were set up for each group, and different concentrations of nicotine (10 μM, 20 μM, 40 μM, 80 μM, 160 μM) were added to the drug-treated wells and incubated at 37C to detect LDH release. One hour before the scheduled assay time, LDH release reagent was added to the most toxic wells in an amount of 10% (25 μL) of the total system and incubated at 37C. After incubation, platelet counts were performed, followed by centrifugation at 480 *g*, and the supernatant from each well was added to a 96-well plate with Lactate dehydrogenase (LDH) assay workup. Incubate for 30 min at room temperature away from light and shake slowly using a horizontal shaker, followed by absorbance measurement at 490 nm and dual-wavelength measurement using 600 nm as the reference wavelength. Finally, mortality was calculated based on the actual absorbance of each well minus the absorbance of the background blank wells, and the cytotoxicity curve was plotted, where the vertical coordinate was the actual absorbance and the horizontal coordinate was the drug concentration.

### 2.6 Platelets aggregation and ATP release assay

Platelet aggregation and ATP release tests were conducted using an aggregometer (Chrono-Log, Havertown, PA), following established protocols. The washed platelets (3 × 10^8^ platelets/mL) (Zhu et al., 2022) were then preincubated with 1 mM of CaCl_2_ and normal saline (as vehicle control), specific inhibitors or varying concentrations of nicotine (10 μM, 20 μM, 50 μM) for 5 minutes while being stirred at 1000 RPM at 37°C. Subsequently, the platelets were activated with different agonists. ATP release from the platelets was quantified using a luciferin-luciferase reagent (Final concentration of 1 μM, Chrono-lume, Chrono-Log). The aggregation and ATP release data were meticulously recorded and analyzed with the help of aggregolink software (Chrono-Log).

### 2.7 Platelet adhesion and spreading on immobilized fibrinogen

Washed platelets were brought to a concentration of 2 × 10^7^ platelets/mL (Song et al., 2019) and subsequently positioned on 24-well tissue culture slides that had been pre-coated with fibrinogen (25 μg/mL) or with bovine serum albumin (as negative control). Subsequently, 2.5 μL of CaCl_2_ (final concentration 1 mM Ca^2+^) and 2.5 μL of MnCl_2_ (final concentration 1 mM Mn^2+^) were added. According to the predetermined grouping, an equal volume of solvent control or nicotine (10 μM, 20 μM, 50 μM) was added to each group. These slides were then placed in a humidified incubator, maintained at 37°C Celsius and a CO_2_ level of 5% for a spell of 45 min. Following a couple of rinses with phosphate-buffered saline, the attached platelets were secured using 2% paraformaldehyde solution. They underwent further processing with a 0.1% Triton X-100 treatment before being dyed with TRITC-conjugated phalloidin (Final concentration of 1 μg/mL). Fluorescent imagery of the specimens was obtained with an Olympus fluorescence microscope at a magnification of ×100, hailing from Japan, and the images were later scrutinized using ImageJ software to gauge the mean coverage area of the spread platelets and the size of individual platelets that had attached.

### 2.8 Clot retraction

A suspension of washed platelets, at a concentration of 500 μL and containing 3 × 10^8^ platelets/mL (Zhu et al., 2022), was compounded. This mixture included 400 μg of 400 μg/mL fibrinogen and 1 mM CaCl_2_. The mixture was pre-treated with different nicotine concentrations (10 μM, 20 μM, 50 μM) or a vehicle control (saline) for 5 min. Platelets without agonist were used as a negative control. The clot contraction process was initiated by introducing 0.2 U/mL of thrombin, after which the mixture was placed in an incubator set at 37°C Celsius and took photos of the samples at 0, 15, 30, and 60 min.

### 2.9 Flow cytometric analysis

Washed platelets (5 × 10^7^ platelets/mL) (Song et al., 2019) were initially pretreated with varying concentrations of nicotine (10 μM, 20 μM, 50 μM), specific inhibitors or vehicle control (saline) for 5 min prior to being activated with thrombin (0.08 U/mL) for another 5 min at 37°C. Following this activation, the platelets were incubated at room temperature, in the dark, for 15 min with either FITC-conjugated anti-P-selectin (CD62P, Final concentration of 1 μM) or FITC-conjugated anti-PAC-1 (CD41/CD61, Final concentration of 1 μM) antibody. The analysis involved counting 10,000 events within the specified population using flow cytometry, and the data were processed using a BD Bioscience flow cytometer.

### 2.10 Calcium signaling assay

Washed human platelets (5×10^7^ cells/mL) (Song et al., 2019) labeled with Fluo-3 AM (Final concentration of 1 μM) were measured using flow cytometry, and platelets were incubated with vehicle control (saline) or nicotine for 5 min 50 μL of platelets after incubation with the drug were taken in a flow tube, and the reaction was terminated by adding 450 μL Buffer. The samples were mixed well and detected by BD flow cytometer, and after the baseline was smooth for about 20 s, 5 μL 10 mM CaCl_2_ (final concentration 1 mM Ca^2+^) and agonist were added and mixed well and the detection was continued for 4 min. The average fluorescence intensity of calcium ions at each 20s time point was analyzed by using the FlowJo software, and the curves were fitted by using the Sigmaplot software.

### 2.11 Western blot

The platelets were treated with 2 × lysis buffer to extract the desired material. The supernatant obtained after centrifugation was assessed for protein concentration using a BCA protein assay kit. The proteins were then separated via 10% SDS-PAGE, transferred onto polyvinylidene difluoride membranes, and subjected to primary and secondary antibody incubation. The bands were ultimately detected using an ECL kit and quantified using ImageJ software.

### 2.12 Statistical analysis

The statistical analysis was conducted using GraphPad Prism 10, a software developed by GraphPad in the United States. The mean values were presented alongside the standard error of the mean (SEM) for every set of data. The student's t-test was employed to compare two groups, while the one-way ANOVA was used to compare multiple groups. Any *p*-value that was less than 0.05 was deemed statistically significant.

## 3 Result

### 3.1 Procoagulant effects of nicotine

**FIGURE 2:**
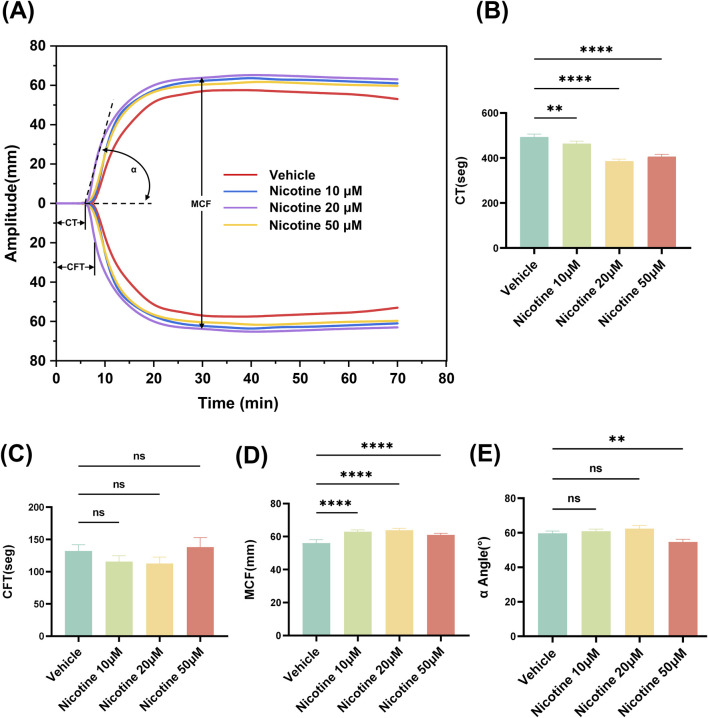
Effect of nicotine on whole blood thromboelastography. **(A)** Representative thromboelastograms for different concentrations of nicotine interventions. Nicotine was added to whole blood over the concentration range shown in the Fig. Effect of nicotine on different parameters of thromboelastograms **(B)** CT, clotting time; **(C)** CFT, clot formation time; **(D)** MCF, maximum clot hardness; **(E)** α-angle. values are expressed as the mean ± SEM of 5 replicates of the experiment and represent the results of 5 independent experiments. One-way ANOVA with Tukey’s multiple comparison. (NS, not significant, * *p < 0.01, * * * *p < 0.0001, vs. vehicle).

Thromboelastometry is a valuable tool for monitoring changes in coagulation (except for vascular endothelial factors). Our study aimed to explore the effects of varying concentrations of nicotine on clot strength and timing, specifically looking at the rate of fibrin formation, clot strength, and clot stability. By examining representative Thromboelastometry at different nicotine concentrations, we were able to observe the procoagulant effect of nicotine (Figure 2A). Interestingly, the addition of nicotine shortened clotting time (CT), with the most notable effect seen at a concentration of 20 μM (*p* < 0.0001, Figure 2B). While there was a tendency for 10 μM and 20 μM nicotine to shorten coagulation formation time (CFT) without significant difference. However, a concentration of up to 50 μM did lead to a prolonging of coagulation time (Figure 2C). The selected concentration of nicotine had a significant impact on maximum coagulation firmness (MCF), with 20 μM nicotine having the most positive effect (*p* < 0.0001, Figure 2D). Notably, there was no significant effect on α Angle (Figure 2E). The most significant differences were observed in the MCF values, which reflect platelet function. As such, our study focused on the effects of nicotine on platelet function.

### 3.2 Nicotine promotes platelet aggregation and release

**FIGURE 3:**
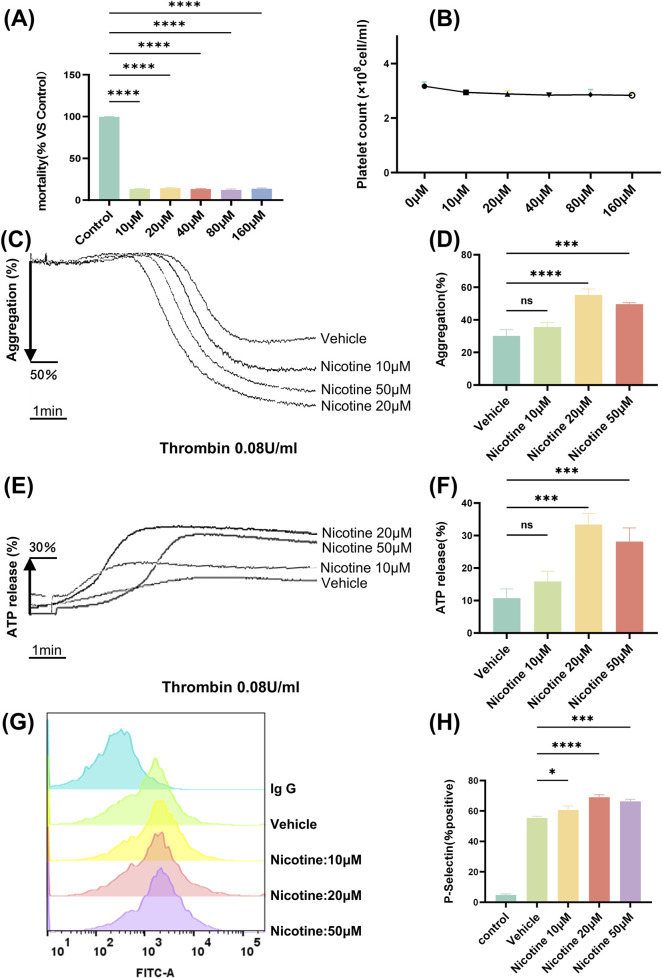
Nicotine promotes platelet aggregation and release. **(A)** Platelet LDH toxicity release assay to assess the effect of nicotine on platelet mortality. **(B)** Effect of different concentrations of nicotine on the number of washed platelets. **(C)** The maximum platelet aggregation rate was determined using a platelet aggregometer following the incubation of washed platelets with varying concentrations of nicotine and stimulation with thrombin. **(D)** Statistical results of platelet aggregation. **(E)** Platelets were treated with different concentrations of nicotine, stimulated with thrombin, and ATP secretion was recorded and quantified. **(F)** Statistical results of ATP release. **(G)** Washed human platelet suspensions were preincubated with nicotine (10, 20, 50 μM) or vehicle, followed by the addition of FITC-labeled P-Selectin, and subsequently stimulated by the addition of thrombin (0.08U), and P-Selectin expression was detected by flow cytometry. **(H)** Statistical results of P-Selectin expression. Values are expressed as the mean ± SEM of 5 replicates of the experiment and represent the results of 5 independent experiments. One-way ANOVA with Tukey’s multiple comparison. (NS, not significant, *p < 0.05, * * *p < 0.001, * * * *p < 0.0001, vs. vehicle).

Aimed at the poison of various concentrations of nicotine to platelets, exploring the LDH exposure of platelets. LDH leakage is a recognized method for evaluating cytotoxicity, with the level of LDH indicative of the degree of cellular disruption. 10–160 μM concentration, almost had no impact on the cell mortality and number of platelets, and the cell mortality of 160 μM Nicotinethe was 13.84% ± 0.4% (VS control *p <* 0.0001, Figures 3A, B).

Platelet aggregation is the experiment that best reflects the function of platelets. The aggregometer reflects the degree of platelet aggregation by detecting the change in turbidity after platelet aggregation. We observed that nicotine alone did not induce platelet aggregation (Supplementary Figure S1). To investigate further, we tested washed platelet aggregation was induced with different agonists (Thrombin, Arachidonic Acid, Collagen), and it was found that nicotine did not significantly promote Arachidonic Acid and Collagen-induced aggregation (Supplementary Figure S2). Whereas, aggregation of washed platelets incubated with a concentration gradient (10 μM, 20 μM, 50 μM) of nicotine, was induced by thrombin at a final concentration of 0.08 U (Figure 3C). Statistical analysis showed that nicotine promoted platelet aggregation, and although there was no difference at a nicotine concentration of 10 μM, both 20 μM and 50 μM nicotine significantly promoted platelet aggregation, and the effect was most pronounced at a concentration of 20 μM (VS control p < 0.0001, Figure 3D). Similarly, to examine the effects of different physiological environments on platelet aggregation, we induced platelet-rich plasma aggregation using various agonists, including thrombin, ADP, arachidonic acid, and collagen. The results demonstrated that nicotine enhanced thrombin-induced platelet-rich plasma aggregation, while it had no significant effect on aggregation induced by the other agonists (Supplementary Figure S3).

By analyzing ATP release (the marker of dense granule) (Figure 3E), we discovered that nicotine enhanced ATP release to the maximum extent at a concentration of 20 μM (VS control *p* < 0.05; 69.03% ± 1.65% after 20 μM nicotine incubation, *p* < 0.0001; 66.37% ± 1.32% after 50 μM nicotine incubation, *p* < 0.001. (Figure 3H). The median fluorescence intensity (MFI) of P-Selectin expression increased significantly, from 1,057 ± 38.34 in the vehicle group to 2,354 ± 32.17 in the 20 μM nicotine group, *p* < 0.0001 (Supplementary Figure S4).

### 3.3 Nicotine promotes platelet integrin receptor αIIbβ3 activation

**FIGURE 4:**
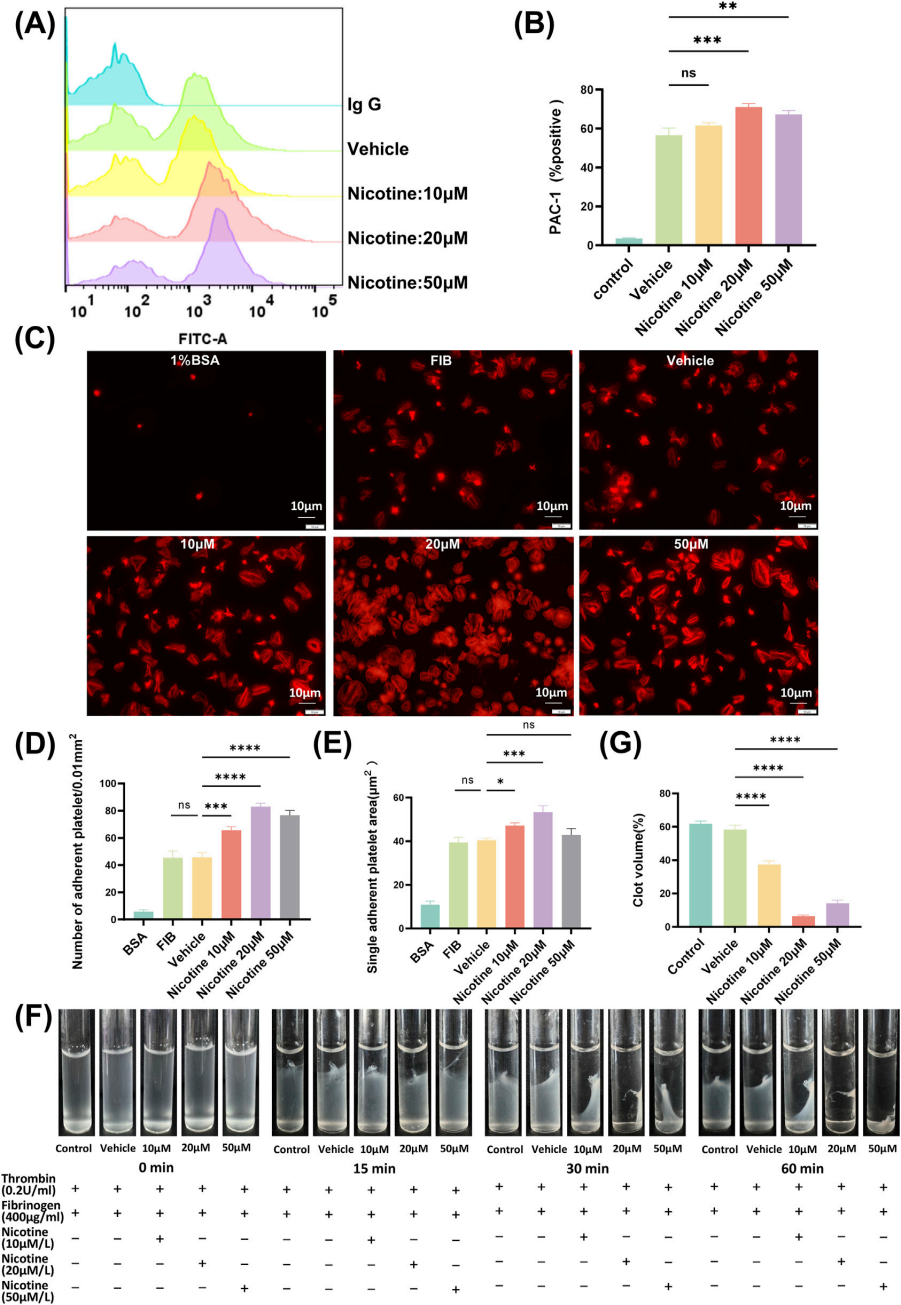
Nicotine promotes platelet integrin receptor αIIbβ3 activation, affecting both “inside-out” and “outside-in” signaling pathways. **(A)** Detection of PAC-1 expression by flow cytometry. Washed human platelet suspensions were preincubated with nicotine (10, 20, 50 μM) or vehicle, and then FITC-labeled PAC-1 was added, followed by thrombin (0.08U) stimulation, and PAC-1 expression was detected by flow cytometry. **(B)** Statistical results of PAC-1 expression. **(C)** The impact of nicotine on platelet adhesion and spreading was assessed through the use of TRITC-tagged ghost pen cyclic peptide dye. The data was crunched by tallying the instances of platelet attachments and averaging out the surface area occupied by each platelet. **(D)** Statistical data based on platelet adhesion numbers. **(E)** An average was derived from the measurements of the expanded surface area for each platelet. **(F)** Visual documentation was captured of clot contraction in response to thrombin stimulation. **(G)** Statistical plot of the percentage of platelet plaques at the 60-min time point. Values are expressed as the mean ± SEM of 5 replicates of the experiment and represent the results of 5 independent experiments. One-way ANOVA with Tukey’s multiple comparison. (NS, not significant, *p < 0.05, * *p < 0.01, * * *p < 0.001, * * * *p < 0.0001, vs. vehicle).

Platelets exhibit an “inside-out” signaling mechanism that agonist stimulation triggers a series of internal signaling events in platelets, such as rearrangement of the platelet backbone, changes in morphology, protein synthesis, and release of granules. Ultimately, this leads to a change in the affinity of the platelet integrin receptor αIIbβ3 for fibrinogen, transitioning from a low to high-affinity state. In humans, the high-affinity state of αIIbβ3 can be detected through the use of PAC-1 antibody, and the activation of integrin αIIbβ3 can be detected by measuring the binding strength of PAC-1 via flow cytometry (Figure 4A). Results from the study showed that final concentrations of 20 μM and 50 μM nicotine led to an increase in PAC-1 expression, while 10 μM nicotine tended to promote PAC-1 expression, but no significant difference was observed (Figure 4B). The MFI of PAC-1 expression demonstrated a significant increase, rising from 354.3 ± 9.597 in the vehicle group to 1741 ± 29.14 in the 20 μM nicotine group, *p* < 0.0001 (Supplementary Figure S4). Platelet activation is primarily driven by the integrin αIIbβ3, which facilitates platelet signaling from the “outside-in” signaling mechanism. To examine the impact of nicotine on platelet adhesion and expansion, platelets were exposed to varying concentrations of nicotine, 1% BSA (as a blank control), fibrinogen (as a Negative control) and vehicle (as a solvent control) (Figure 4C). The staining of the platelet skeleton using ghost pen cyclic peptides showed a significant rise in platelet adhesion and spreading on fibrinogen among the nicotine-treated group across all tested concentrations, and this promotion was not attributable to the solvent. The most striking variation emerged at 20 μM, with the number of adhering platelets increasing from 49.11 ± 2.30 platelets/0.01 mm^2^ to 86.12 ± 2.14 platelets/0.01 mm^2^. Additionally, there was a noticeable growth in the average expansion area of individual platelet, which increased from 49.87 ± 1.59 μm^2^ to 54.07 ± 1.08 μm^2^ (Figures 4D, E). These results suggest that nicotine promotes platelet adhesion and expansion on fibrinogen. Thromboconstriction occurs following primary and secondary hemostasis. The findings from the plaque retraction assay demonstrated that all three concentrations of nicotine facilitated the contraction of platelet plugs after 60 min. Notably, the 20 μM concentration of nicotine exerted the most pronounced promotional effect (Figures 4F, G).

### 3.4 Nicotine promotes thrombin-induced increase in platelet intracellular Ca^2+^ concentration

**FIGURE 5:**
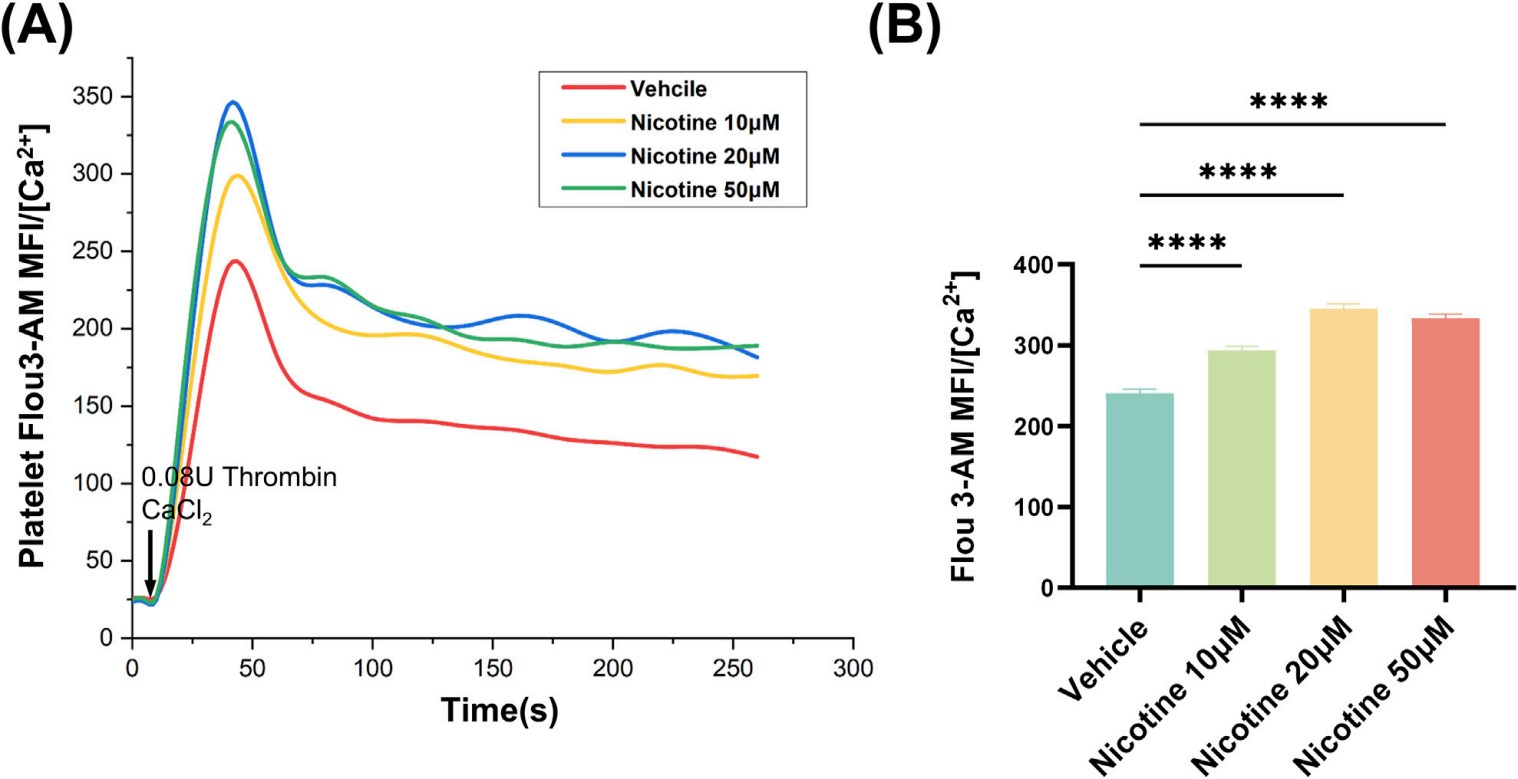
Nicotine promotes thrombin-induced increases in platelet intracellular Ca^2+^ concentration. **(A)** 20s for basal intraplate-let calcium levels. Platelets were then stimulated with Thrombin and Ca^2+^ to detect the effect of nicotine on the release of intracellular calcium stores from platelets. **(B)** Statistics of intracellular Ca^2+^ concentration kurtosis in platelets under the intervention of nicotine at the concentrations shown. Values are expressed as the mean ± SEM of 5 replicates of the experiment and represent the results of 5 independent experiments. One-way ANOVA with Tukey’s multiple comparison. (* * * *p < 0.0001, vs. vehicle).

The signaling of platelet intracellular calcium ions plays a critical role in the activation of platelets. Upon activation, agonists cause an increase in the concentration of intracellular calcium ions within platelets. This heightened concentration is a result of the release of intracellular calcium pools, as well as the inward flow of extracellular calcium ions. By utilizing the Flou-3-AM dye to bind to free intracellular calcium ions and measuring the resulting fluorescence intensity through flow cytometry, changes in platelet intracellular calcium ion concentration due to nicotine were detected (Figure 5A). All concentration gradients of nicotine were found to promote an increase in intracellular calcium ion concentration in platelets, with the highest concentration observed at 20 μM nicotine (Figure 5B).

### 3.5 Nicotine is a potential agonist of the platelet PAR4 receptor

**FIGURE 6:**
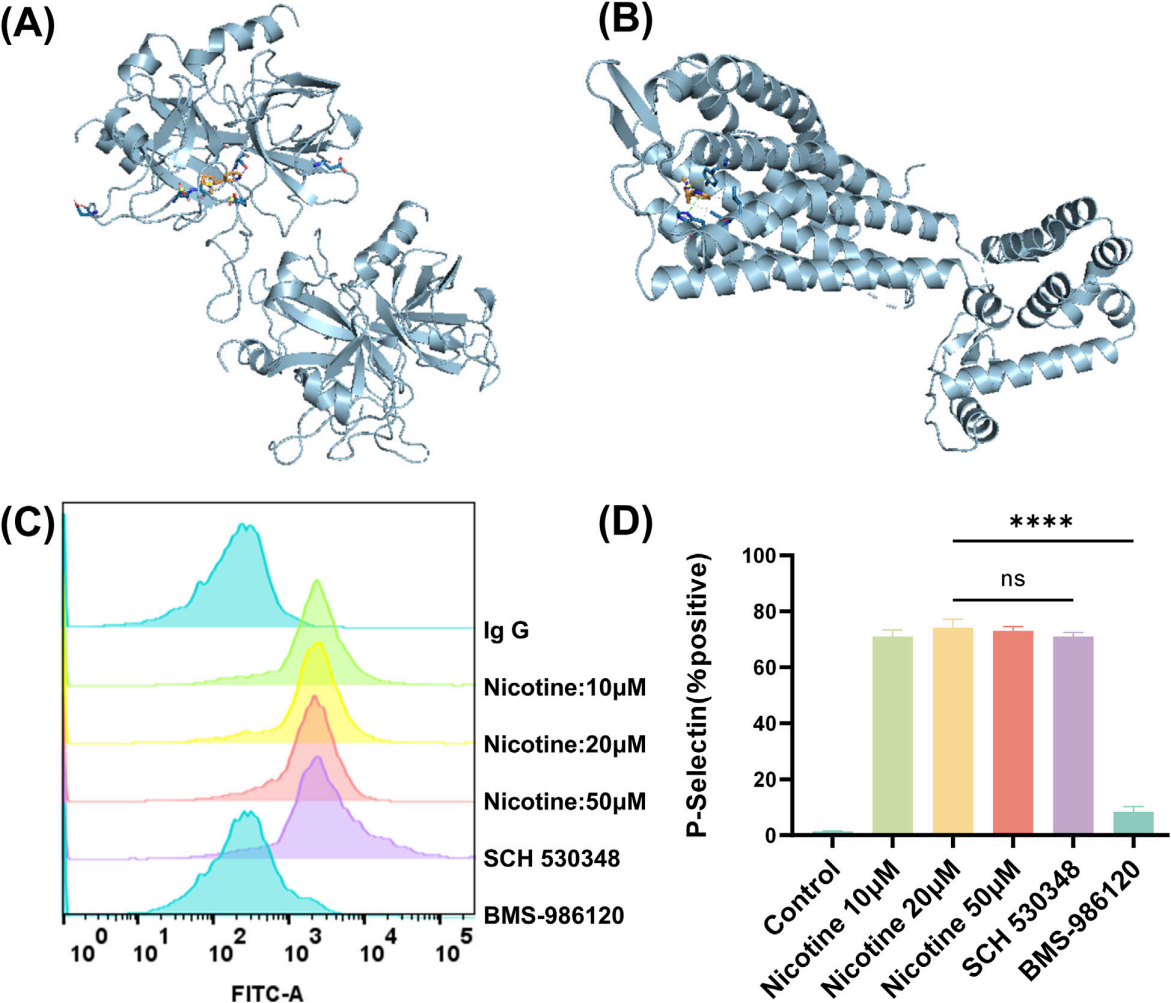
Nicotine is a potential agonist of the platelet PAR4 receptor. Prediction of nicotine-bound target receptors on platelet membranes using molecular docking simulation software. **(A)** Interaction of nicotine with PAR1 in 3D. **(B)** Interaction of nicotine with PAR4 in 3D. **(C)** Effect of nicotine and PAR1 and PAR4 inhibitors on P-Selectin positive expression. **(D)** Statistical results of P-Selectin positive expression. Values are expressed as the mean ± SEM of 5 replicates of the experiment and represent the results of 5 independent experiments. One-way ANOVA with Tukey’s multiple comparison. (NS, not significant, * * * *p < 0.0001, vs. vehicle).

To determine which receptors on platelet membranes were bound by nicotine, utilizing molecular docking simulation software. This method involves creating a simulation of the forces that occur between receptors and ligands, and binding them together in a way that maximizes their complementary shapes and properties. The results showed that nicotine had a stronger binding score with PAR1, PAR4 receptors. Molecular docking was used to map the interaction of nicotine with two receptors, PAR1 and PAR4, visualizing through a schematic of the 3D interaction (Figures 6A, B). Additionally, the flow cytometry assay demonstrated that nicotine alone could activate platelet P-Selectin expression (Figure 6C), indicating the release of α-granules. Importantly, the facilitation of this process could not be reversed by SCH 530348 (PAR1 receptor inhibitor), but could be reversed by BMS-986120 (PAR4 receptor inhibitor) (Figure 6D). The MFI of P-Selectin was measured at 1775 ± 21.94 following the administration of SCH 530348, which did not show a significant reversal compared to the 20 μM nicotine group (1802 ± 19.13). In contrast, the MFI of P-Selectin significantly decreased to 120.3 ± 7.68 with the application of BMS-986120 (Supplementary Figure S4). Furthermore, BMS-986120 effectively countered the pro-aggregation effect of nicotine in the thrombin-induced platelet-rich plasma aggregation assay, while SCH 530348 was unable to produce a similar reversal (Supplementary Figure S5).

### 3.6 Nicotine specifically activates the PAR4 downstream signaling pathway

**FIGURE 7:**
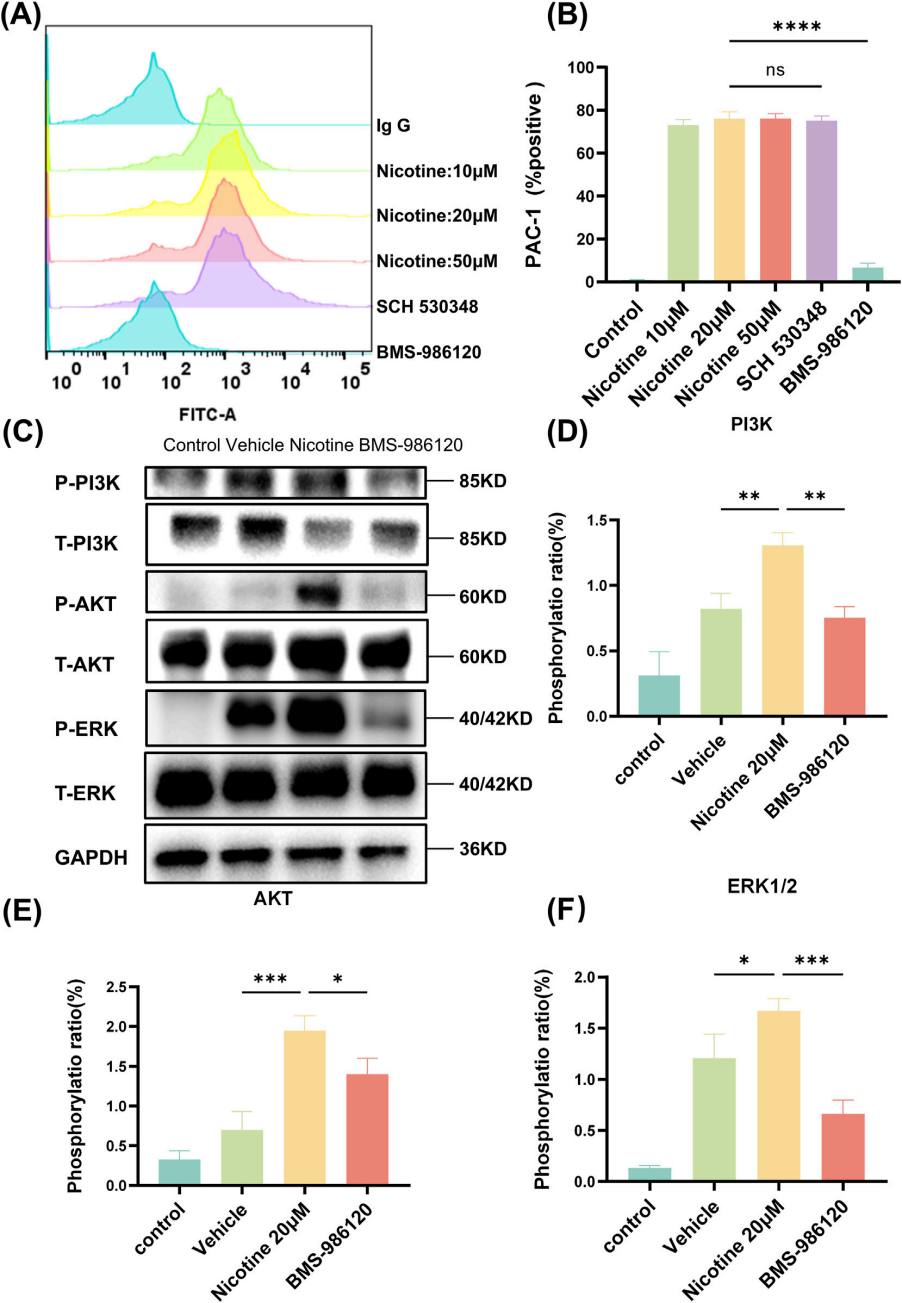
Nicotine specifically activates the PAR4 downstream signaling pathway. **(A)** The impact of nicotine and PAR1 and PAR4 inhibitors on PAC-1 expression. **(B)** The statistical findings related to PAC-1 expression. **(C)** Nicotine induces the phosphorylation of PAR4 downstream signaling molecules, leading to aIIbβ3 activation. Platelets were treated with nicotine until overall aggregation was achieved, after which they were lysed, and the levels of signaling molecules were measured. **(D–F)** Western blot analysis was conducted to observe the influence of nicotine on the levels of phosphorylation of PI3K, AKT, and ERK1/2. Values are expressed as the mean ± SEM of 5 replicates of the experiment and represent the results of 5 independent experiments. One-way ANOVA with Tukey’s multiple comparison. (NS, not significant, *p < 0.05, * * *p < 0.01, * * * *p < 0.001, * * * * *p < 0.0001, vs. vehicle).

Upon platelet activation, a series of intracellular signal transduction events converge on the cytoplasmic tail of αIIbβ3, causing the extracellular structural domain of αIIbβ3 to transition to a high-affinity state for its extracellular ligand. The activation status of αIIbβ3 was assessed via flow cytometry (Figure 7A), and it was found that nicotine alone induced the activation of the platelet integrin receptor αIIbβ3, a state that could only be reversed by BMS-986120 (Figure 7B). The MFI of PAC-1 was 876.7 ± 3.71 following treatment with SCH 530348, showing no significant reversal compared to the 20 μM nicotine group (890 ± 5.56). In contrast, the MFI of P-Selectin decreased markedly to 13.67 ± 1.45 after treatment with BMS-986120 (Supplementary Figure S4). To delve deeper into the impact of nicotine on these targets, Western blotting was utilized to gauge the phosphorylation levels of specific proteins (Figure 7C). The findings revealed a significant increase in the phosphorylation levels of PI3K (Figure 7D), AKT (Figure 7E), and ERK1/2 (Figure 7F) induced by nicotine.

## 4 Discussion

Nicotine, the primary active component in tobacco, has various pharmacological effects and promotes platelet activity (Renaud et al., 1984; Qasim et al., 2017; Deng et al., 2024). Investigating the impact of nicotine on platelet function and its mechanism could support its potential use as a local hemostatic agent. Our research shows that nicotine significantly improves platelet function, affecting aggregation, granule release, adhesion, spreading, and clot retraction. Additionally, nicotine stimulates platelet activation via PAR4 receptors, leading to increased intracellular calcium ion levels and the phosphorylation of signaling molecules downstream of PAR4 receptors. Nicotine’s mechanisms of action in promoting platelet function include the following: 1. Enhancement of thrombin-induced elevation of platelet intracellular calcium concentration; 2. Facilitation of integrin αIIbβ3 ″inside-out” and “outside-in” signaling; 3. Stimulation of phosphorylation of signaling pathways downstream of the PAR4 receptor (PI3K-AKT, ERK-1/2). In a review by Zlabek, it was established that nicotine induces oxidative stress, which subsequently promotes abnormal platelet function (Ford and Zlabek, 2005). Ljungberg et al. examined the impact of various nicotine concentrations on ADP-induced platelet aggregation (Ljungberg et al., 2013). However, these studies primarily identified the phenomenon without delving deeply into its underlying mechanisms, and our study aims to address this gap. Our findings support previous research on nicotine’s role in signaling pathways, improving our understanding of its physiological effects, particularly on platelet function, and suggesting its potential as a topical hemostatic agent.

Physiological hemostasis involves vasoconstriction, platelet aggregation, thrombosis, and blood coagulation (Wu et al., 2014). When blood vessels are damaged, platelets stick to the vessel wall, release substances that cause vasoconstriction, and activate the coagulation system, leading to a quick hemostatic response (Li et al., 2010). Initially, we employed thromboelastography, which provided comprehensive data on clot formation and strength (Nogami, 2016), demonstrating that nicotine exerts procoagulant and hemostatic effects through its influence on platelet function. The MCF value serves as a key indicator of the strength and stability of thrombus formation, reflecting the aggregative capacity of platelets (Azarfarin et al., 2018). An increase in the MCF value demonstrated that platelets are more extensively aggregated, resulting in a more robust thrombus formation, which enhances the hemostatic effect. Platelet activation plays a crucial role in both physiological hemostasis and pathological thrombosis (Khodadi, 2020). Thrombin serves as a pivotal enzyme in the blood coagulation process, playing a crucial role in clot formation and hemostasis (Larsen and Hvas, 2021). Its capacity to trigger platelet activation and subsequently facilitate platelet aggregation makes it a preferred platelet agonist in our research. We examined nicotine’s cytotoxicity on platelets and its impact on their activation and function *in vitro*. Based on the findings from the toxicity experiments, along with the results from Ljungberg et al. (2013), we identified 10, 20, and 50 μM as the concentration ranges for our study. By preincubating washed human platelets with nicotine (10, 20, 50 μM), we found that nicotine enhanced platelet aggregation and granule release. Platelet aggregation and granule release are two key processes in the platelet response, playing a vital role in hemostatic function. Nicotine can activate downstream signaling pathways by binding to receptors on the surface of platelets, thereby enhancing both platelet activation and granule release (van der Meijden and Heemskerk, 2019). The administration of 20 μM nicotine significantly enhanced platelet function; however, elevated doses exhibited certain limitations. Further investigation into the effects of varying concentrations of nicotine on platelet function is essential to establish a safe therapeutic range in future studies.

The integrin receptor αIIbβ3 is crucial for platelet hemostasis, transitioning from a low-affinity resting state to a high-affinity activated state during inside-out signaling (Arman et al., 2014). The “inside-out” signaling refers to the activation of the platelet’s internal signaling system, which leads to a conformational change in αIIbβ3, exposing the functional site for binding ligands such as fibrinogen (Ma et al., 2007). The activation state of αIIbβ3 can be evaluated by measuring PAC-1 expression, revealing nicotine’s effect on “inside-out” signaling in platelets. This activated state of αIIbβ3 binds to fibrinogen, triggering a series of intracellular signaling events that converge at the cytoplasmic tail of αIIbβ3, initiating “outside-in” signaling (Reininger, 2008). By enhancing the “outside-in” signaling of platelets, nicotine can exacerbate morphological changes, particularly after binding to fibrinogen, which increases contact with the matrix through morphological alterations and pseudopod extension, thereby enhancing adhesion strength and surface area. The synergy between “inside-out” and “outside-in” signaling promotes crucial platelet processes such as expansion and plaque retraction (Kasirer-Friede et al., 2007). Nicotine was found to promote the activation of platelet integrin receptor αIIbβ3, activating both “inside-out” and “outside-in” signaling in platelets.

Our research has further explored nicotine’s effects on platelet activation and function. The mechanisms underlying platelet activation are diverse, however, they converge on the elevation of intracellular calcium concentrations. Substantial quantities of free Ca^2+^ are sequestered within membrane-bound organelles, notably the dense tubular system (DTS) and acidic compartments such as lysosomes and dense granules (Broos et al., 2012). Nicotine has been found to facilitate elevated intracellular calcium ion concentrations through the release of intracellular calcium pools and inward flow of extracellular calcium ions. Most inducers cause the release of calcium ions from the DTS calcium pool (Chen et al., 2015; Martyanov et al., 2020). Upon the interaction of inositol 1,4,5-trisphosphate (IP3), generated through the phospholipase C (PLC)-mediated hydrolysis of phosphatidylinositol 4,5-bisphosphate (PIP2) situated on the cytosolic membrane of platelets (Bonnet and Tran Van Nhieu, 2016), with the IP3 receptor on the dense tubular system, the receptor undergoes clustering and subsequent activation, leading to the opening of the associated calcium channel (Lencesova and Krizanova, 2012). However, the specific calcium pools and calcium channels associated with this process require further study.

Subsequently, we utilized molecular docking simulation software to forecast nicotine-bound target receptors on platelet membranes. Molecular docking simulations quantitatively assess the interaction energies between molecules, encompassing van der Waals forces, hydrogen bonds, and electrostatic interactions, through the construction of spatial docking configurations involving nicotine and classical receptors on platelet membranes (Singh et al., 2022). These interaction energies serve as indicators of the strength and stability of molecular binding. Notably, the docking scores of nicotine and PAR4 were found to be favourable, and the application of BMS-986120 reversed the activating effect of nicotine on platelets (including positive expression of P-Selectin and PAC-1). This suggests that nicotine impacts platelet function by influencing the downstream signaling pathway of platelet PAR4. PAR4 is known to signal platelet aggregation through calcium mobilization and purinergic signaling pathways (Holinstat et al., 2006; Murugappa and Kunapuli, 2006), with its activation being sufficient to trigger platelet secretion and aggregation (Ml et al., 1999). Furthermore, the activation of PAR4 initiates the downstream signaling of G12/13 and Gq through RhoA, consequently modulating an extensive network of signaling pathways and promoting the release of dense granules. (Coughlin, 2000). The results showed that nicotine activates PAR4 signaling pathways, increasing PI3K, AKT, and ERK1/2 phosphorylation, which suggests that nicotine enhances platelet function and promotes hemostasis. Further investigations into the PAR4 receptor and its downstream signaling pathways are essential to enhance our understanding of its molecular mechanisms during platelet activation. Moreover, studying the role of PAR4 in disease models—particularly in pathological conditions such as atherosclerosis, venous thrombosis, and cancer-associated thrombosis—will be crucial for evaluating its potential as a therapeutic target for intervention.

Despite the significant insights provided by these experimental results regarding the role of nicotine in platelet function, our study is subject to certain limitations. Primarily, the investigation was conducted using an *in vitro* model, which has not yet been validated in an *in vivo* context. Although *in vitro* experiments offer valuable data on platelet function, the complexity and dynamics inherent in the *in vivo* environment may alter the effects of nicotine. Consequently, subsequent research endeavors should prioritize the utilization of *in vivo* models to comprehensively elucidate the role of nicotine in platelet activation and to ascertain its definitive impact on physiological hemostasis.

One critical aspect that warrants attention is the dual role of nicotine in modulating thrombotic risk while promoting hemostasis, particularly in patients at risk for cardiovascular disease. Future studies should focus on elucidating the dose-response relationship between nicotine exposure and platelet activation, with a particular emphasis on acute exposure. Additionally, safety evaluations are paramount when considering nicotine as a potential clinical treatment and its possible adverse effects must be fully considered, specifically in balancing its therapeutic efficacy while minimising the risk of thrombosis and other cardiovascular complications. Long-term nicotine exposure necessitates a comprehensive assessment of its effects on both platelets and the broader cardiovascular system. This includes not only its immediate impact on platelet function but also its chronic effects on vascular health and hemodynamics. To better understand the risks associated with chronic nicotine use, future research should prioritize the development of more robust animal models and clinical trials that systematically evaluate its effects on platelet activity, coagulation, and overall cardiovascular safety (Dai et al., 2022). Further investigation into nicotine’s blood concentration, therapeutic window, and its impact on both the cardiovascular and central nervous systems, particularly in trauma models, is essential. This will help clarify nicotine tolerance across different populations through careful dosage control and provide critical insights into the long-term safety of nicotine use. Ultimately, such studies will be pivotal in ensuring that potential cardiovascular risks are minimized in therapeutic contexts.

In conclusion, this study has substantiated the comprehensive impact of nicotine on platelet function and elucidated the underlying mechanisms. Specifically, this mechanism involves the activation of the thrombin-specific PAR4 receptor signaling pathway. Additionally, nicotine promotes an increase in platelet intracellular calcium ion concentration induced by thrombin. These findings offer valuable insights into the mechanisms by which nicotine facilitates platelet activation and provide important evidence for its potential role as a local hemostatic agent.
