# Supplementary figures and images for: Nicotine’s impact on platelet function: insights into hemostasis mechanisms
Source: Front Pharmacol. 2025 Jan 20;15:1512142. doi: 10.3389/fphar.2024.1512142 (PMC11788582; doi:10.3389/fphar.2024.1512142)

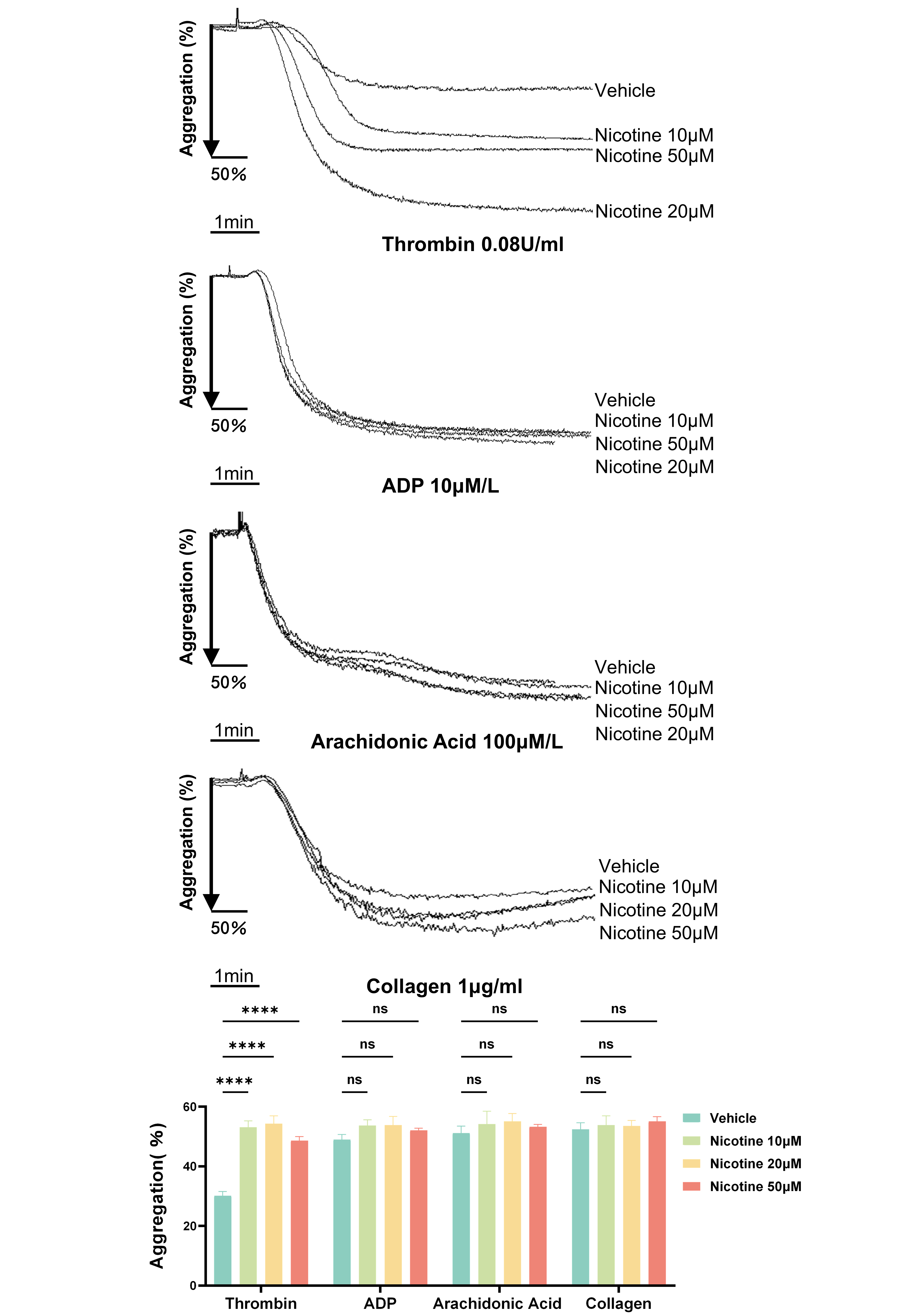

Supplement: Supplementary file 1 [file Image3.tif]

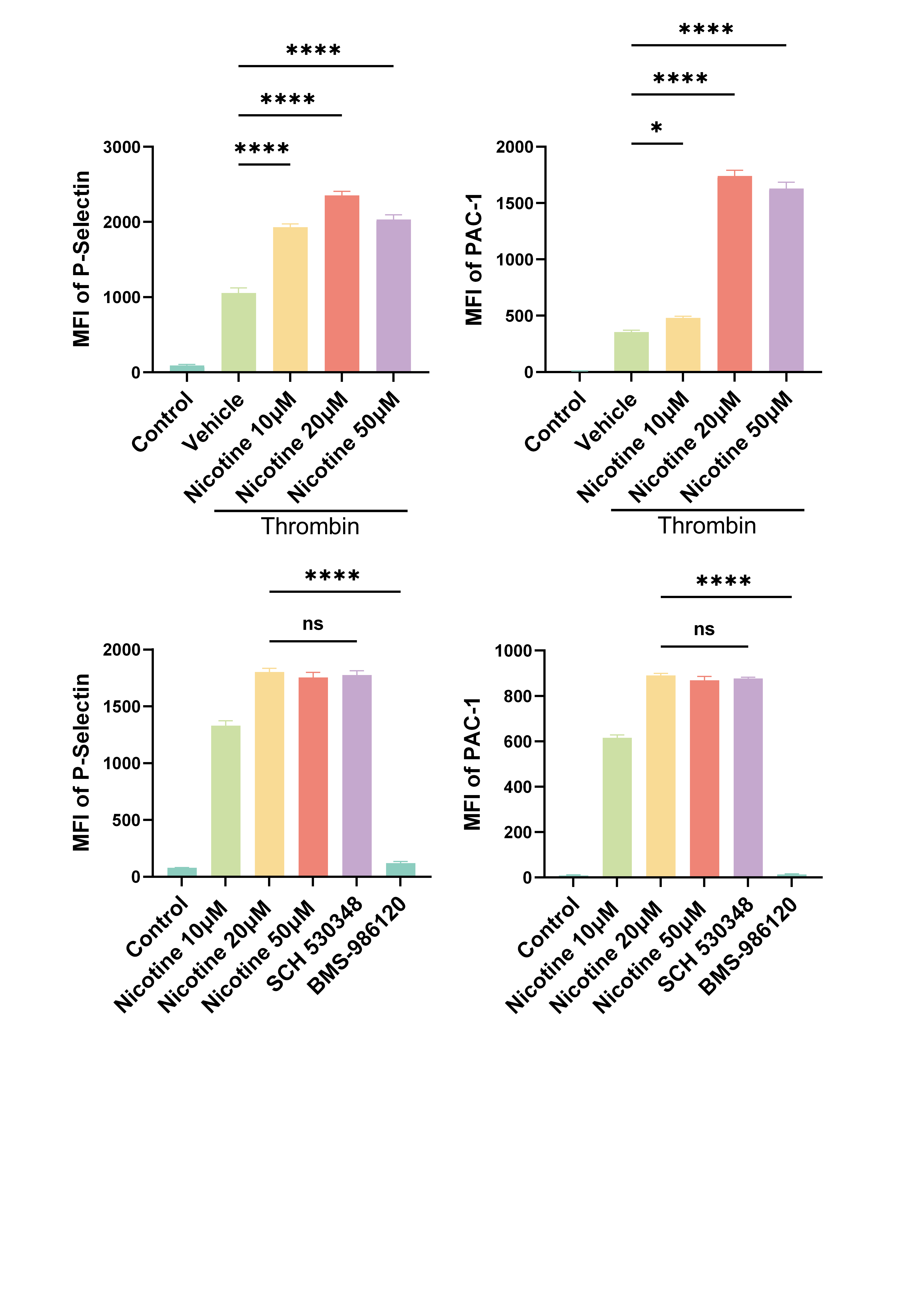

Supplement: Supplementary file 2 [file Image4.tif]

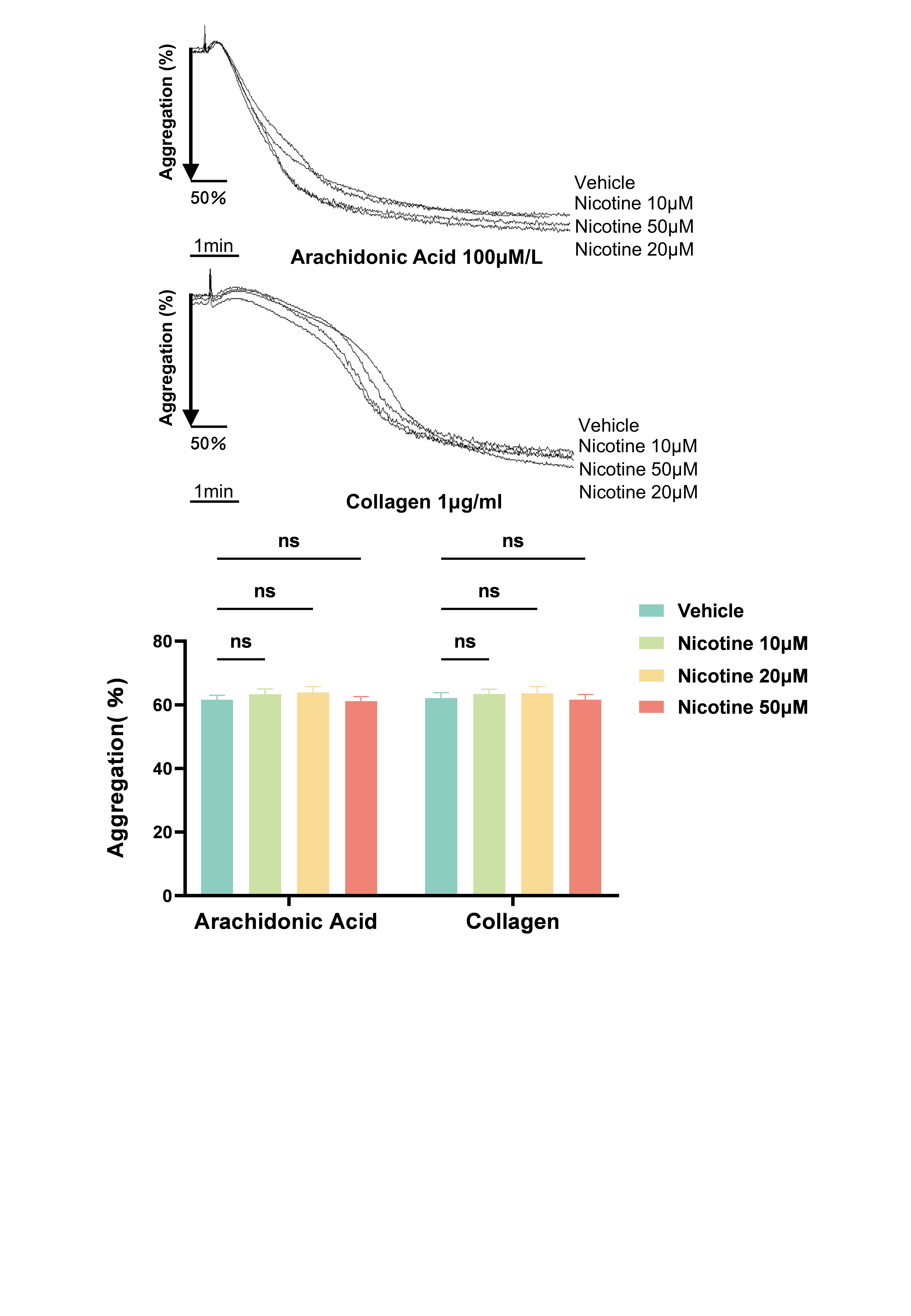

Supplement: Supplementary file 3 [file Image2.tif]

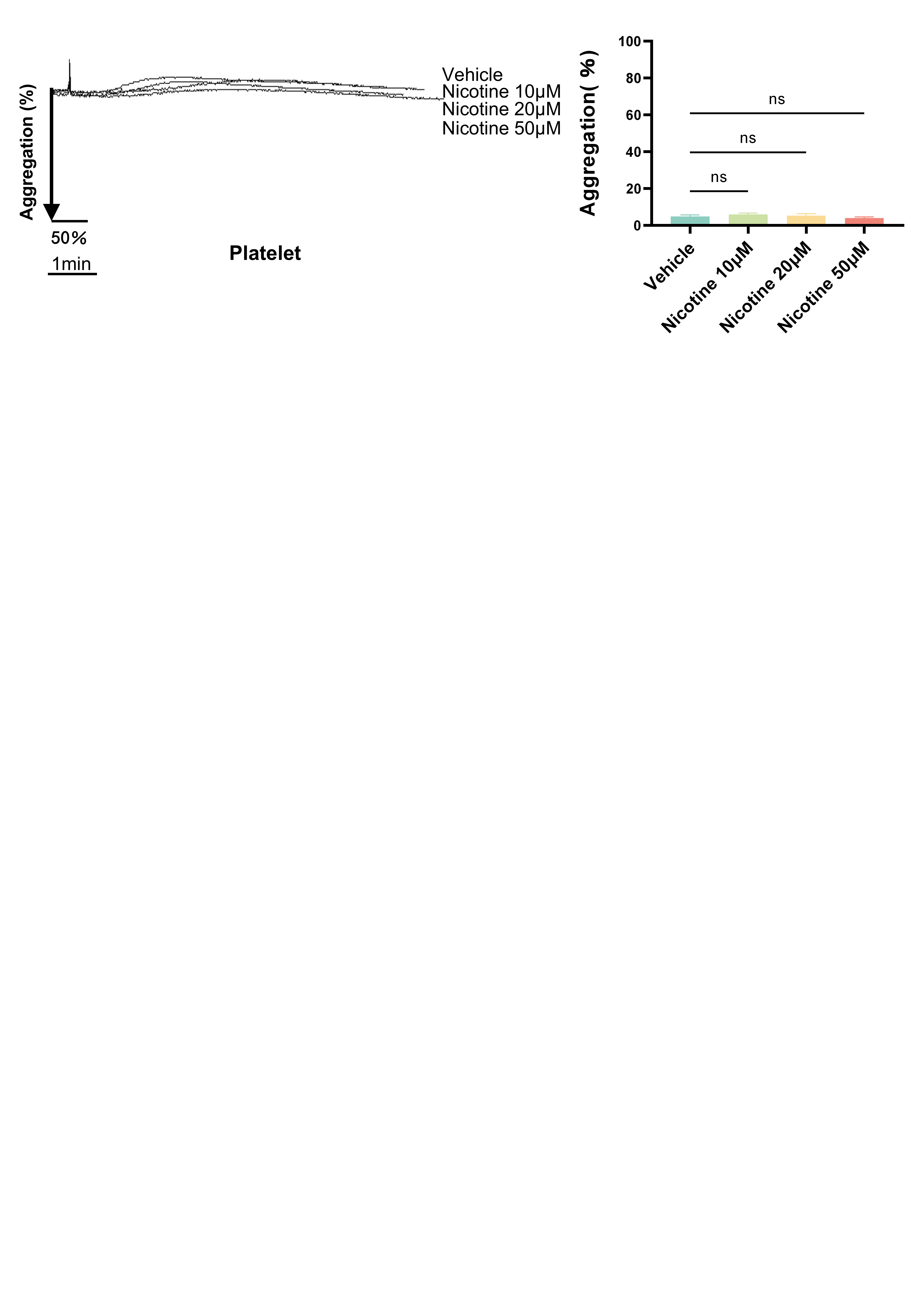

Supplement: Supplementary file 4 [file Image1.tif]

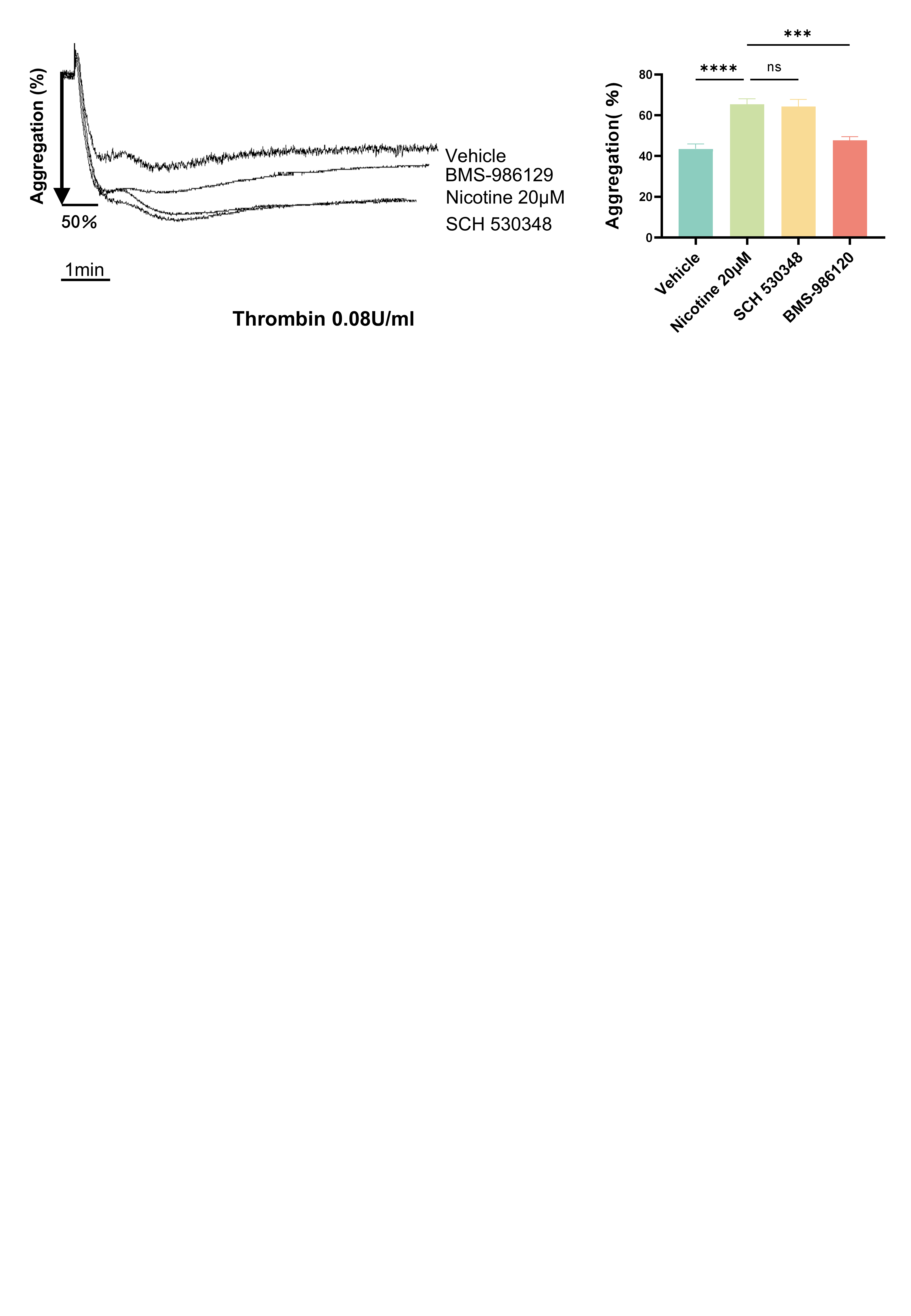

Supplement: Supplementary file 6 [file Image5.tif]
