# Supplementary material for: Nicotine’s impact on platelet function: insights into hemostasis mechanisms
Source: Front Pharmacol. 2025 Jan 20;15:1512142. doi: 10.3389/fphar.2024.1512142 (PMC11788582; doi:10.3389/fphar.2024.1512142)
Supplement: Supplementary file 5 [file DataSheet1.pdf]

Supplementary Material

1 Supplementary Figures and Tables

1.1 Supplementary Figures

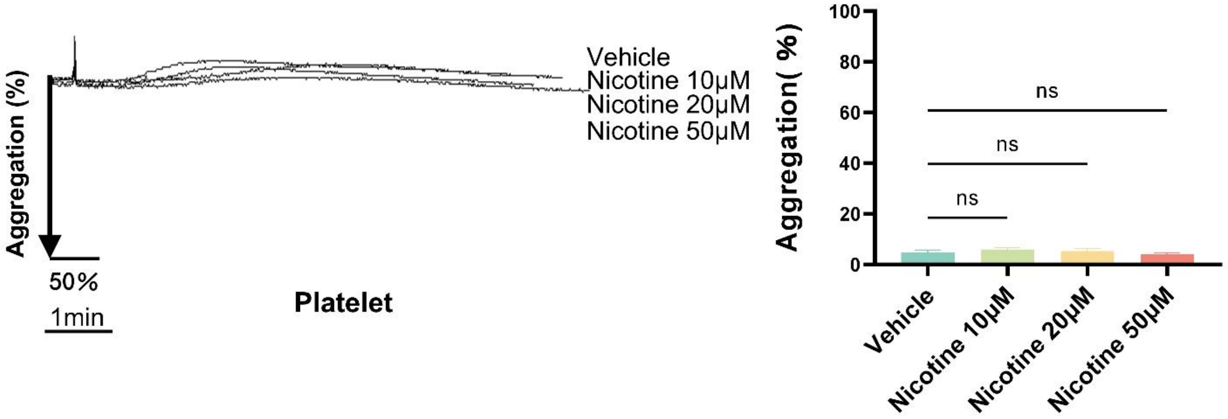

Supplementary Fig S1. Effects of nicotine without agonist on platelet aggregation.

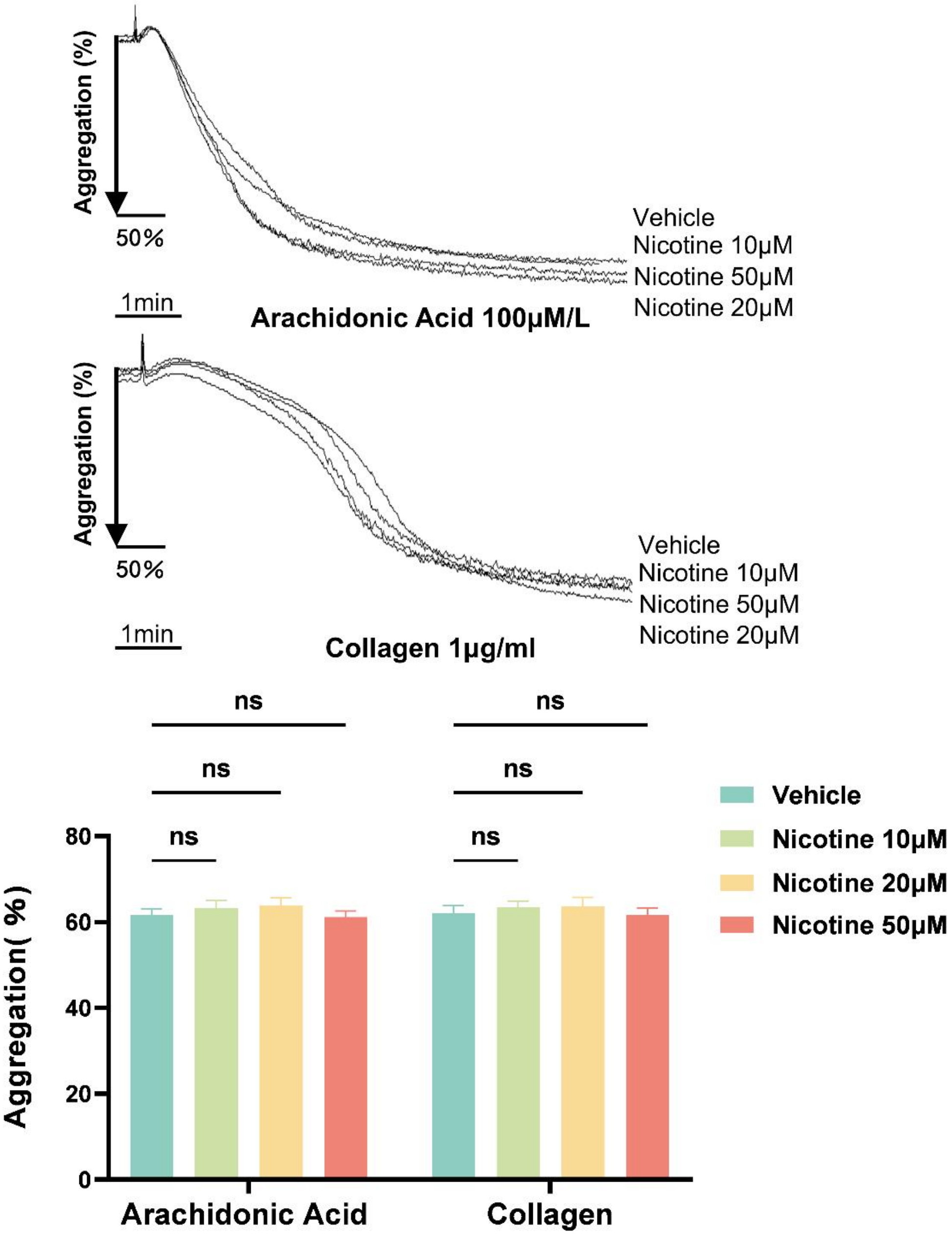

Supplementary Fig S2. Effects of nicotine on washed platelet aggregation induced by different agonists (Arachidonic Acid and Collagen).

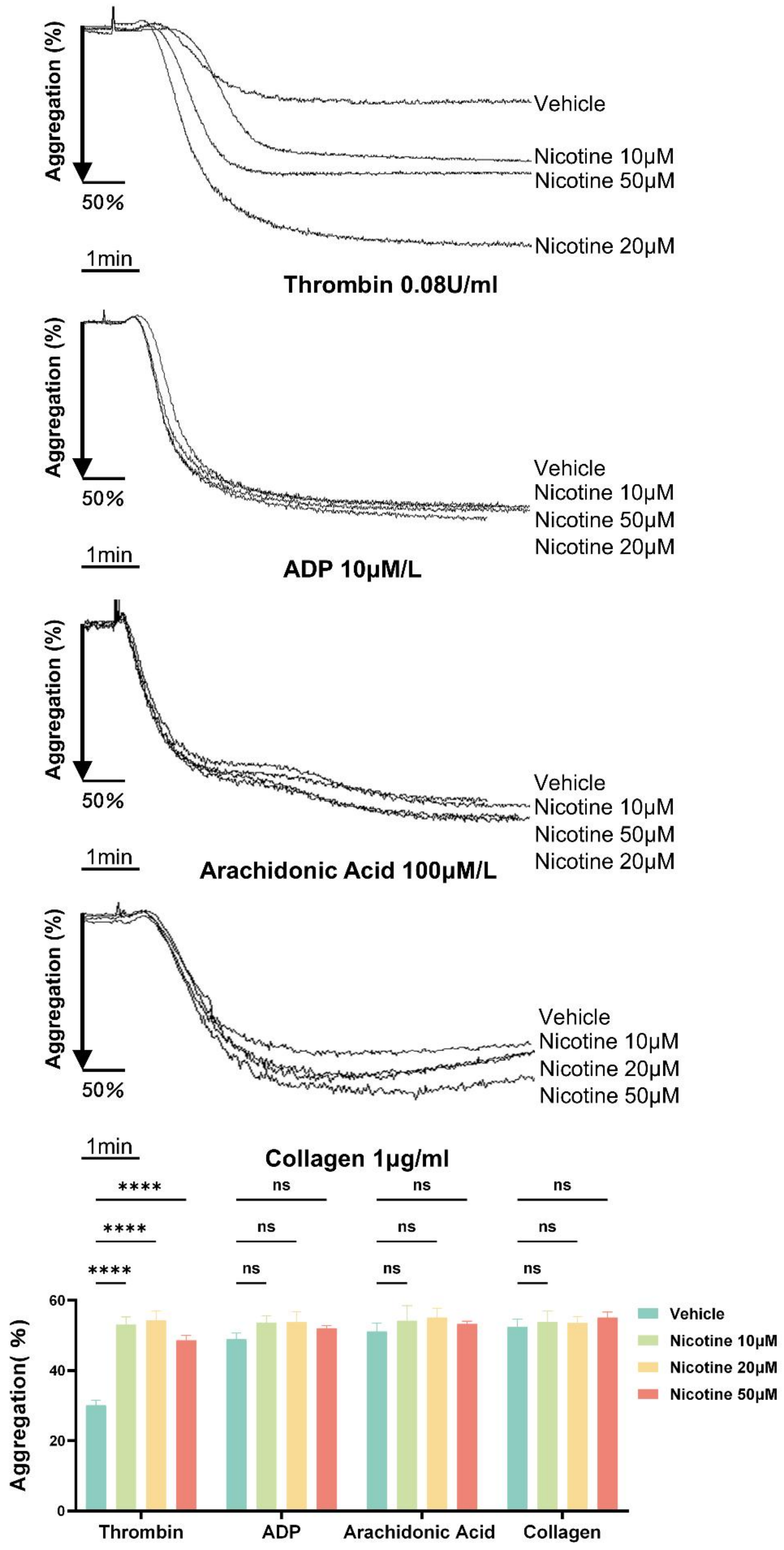

Supplementary Fig S3. Effects of nicotine on platelet-rich plasma aggregation induced by different agonists (Thrombin, ADP, Arachidonic Acid, Collagen).

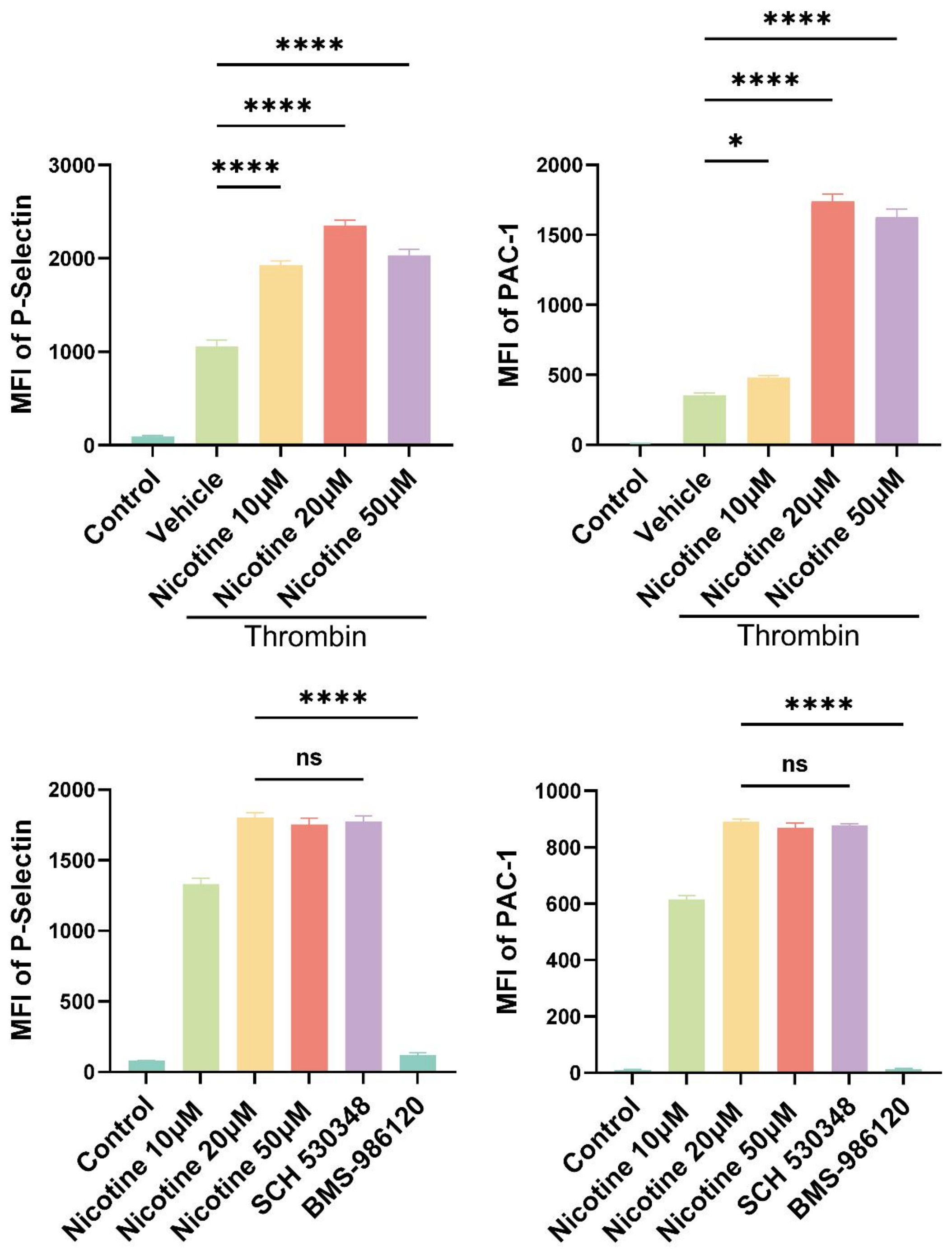

Supplementary Fig S4. Statistics of Platelet P-Selectin and PAC-1 median fluorescence intensity (MFI) Values Determined by Flow Cytometry.

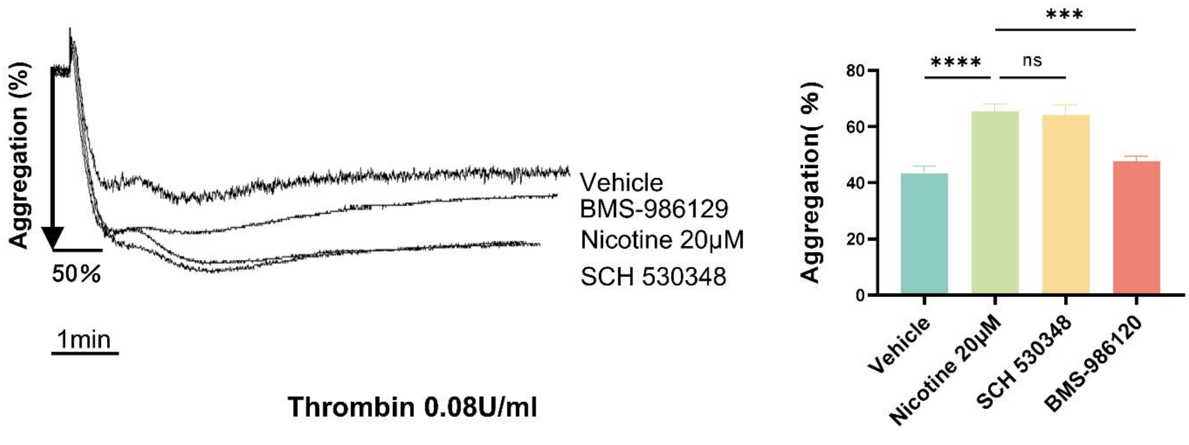

Supplementary Fig S5. Role of PAR1 inhibitor (SCH 530348) and PAR4 inhibitor (BMS-986120) on nicotine enhancement of thrombin-induced platelet-rich plasma aggregation.
